# Comparative Study of Chitosan-Pyrophosphate and Magnesium Hydroxide-Alginate Hybrid Nanoparticles: Physicochemical Properties and Cytocompatibility toward Vascular Calcification Applications

**DOI:** 10.1021/acsomega.5c12883

**Published:** 2026-05-01

**Authors:** Jorge Costa Silva Filho, Maíra Maftoum Costa, Marcelo de Sousa, Laryssa Cristine Ribeiro dos santos, Talita Mazon

**Affiliations:** † 193232 Centro de Tecnologia da Informação Renato Archer, Rodovia D. Pedro I (SP-65), Km 143,6, 13069-901 Bairro Amarais, Campinas, São Paulo , Brazil; ‡ Department of Chemistry, University Federal of Amazonas (UFAM), Av. Rodrigo Otávio, 6.200, 69077-000 Manaus, Amazonas, Brazil

## Abstract

Vascular calcification (VC) is a progressive pathological process that contributes significantly to cardiovascular morbidity, particularly in patients with chronic kidney disease. In this study, we developed and compared two biocompatible nanoparticle formulations designed for biomedical applications in the context of vascular calcification: chitosan-sodium pyrophosphate (NPs-CS-PPi) and magnesium hydroxide-alginate (NPs-Mg­(OH)_2_-Alg). The nanoparticles were synthesized via ionic gelation and hydrothermal precipitation methods, followed by surface functionalization. Comprehensive physicochemical characterization was performed using dynamic light scattering (DLS), Fourier transform infrared spectroscopy, thermogravimetric analysis, X-ray photoelectron spectroscopy, and scanning electron microscopy. Both formulations exhibited spherical morphology, hydrodynamic diameters under 200 nm, and a low polydispersity index (PDI < 0.2). Surface charge measurements indicated successful functionalization, with zeta potentials ranging from +22 mV (NPs-CS-PPi) to −15 mV (NPs-Mg­(OH)_2_-Alg). In vitro cytotoxicity assays using L929 fibroblasts confirmed high biocompatibility, meeting ISO 10993-5 criteria, with cell viability exceeding 90% for NPs-Mg­(OH)_2_-Alg and above 80% for NPs-CS-PPi across all tested concentrations. The magnesium-based system demonstrated a more favorable balance between physicochemical properties and cytocompatibility. Overall, this work establishes a robust physicochemical and cytocompatibility framework that supports future studies employing dedicated vascular calcification and biomaterial evaluation models.

## Graphical abstract

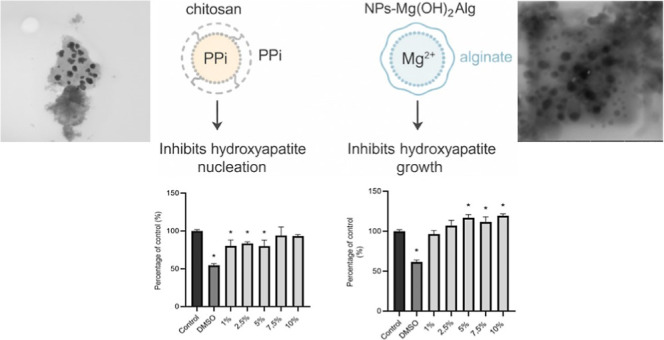

## 1 Introduction

Vascular calcification (VC) is a regulated, cell-mediated pathological process that contributes significantly to cardiovascular morbidity and mortality, particularly among patients with chronic kidney disease (CKD) and diabetes. It is characterized by the pathological deposition of calcium phosphate minerals, primarily hydroxyapatite, within the vascular extracellular matrix, leading to arterial stiffening, reduced elasticity, and an increased risk of cardiovascular events such as myocardial infarction and stroke.^1,2^ VC is not a passive degenerative event but a highly orchestrated process involving molecular pathways such as osteogenesis, in which vascular smooth muscle cells (VSMCs) transdifferentiate into osteoblast-like phenotypes.^3,4^

The development of VC arises from an imbalance between promoters and inhibitors of mineralization. Elevated levels of phosphate, calcium, oxidative stress, and chronic inflammation, frequently observed in CKD, promote the formation and maturation of calciprotein particles (CPPs), which serve as precursors of mineral deposition (3,5). Simultaneously, reductions in physiological inhibitors, such as fetuin-A, osteoprotegerin, Klotho, matrix Gla protein (MGP), and magnesium, exacerbate calcification.^4,5^ Despite therapeutic advances, current interventions that rely on phosphate or calcium control remain insufficient to prevent the progression of vascular mineralization.^6^

Among emerging biomaterial-based strategies, magnesium has gained increasing attention due to its pivotal role in modulating VC. Clinical and experimental data indicate that low serum magnesium levels correlate with increased aortic calcification and cardiovascular mortality in CKD patients.^7,8^ Magnesium acts by inhibiting hydroxyapatite crystal growth, suppressing the osteogenic differentiation of VSMCs, and stabilizing extracellular matrix integrity.^7,9^ Studies in Klotho-deficient animal models that mimic mineral bone disorders of CKD have shown that magnesium supplementation prevents VC, though systemic delivery may cause undesirable side effects.^9^ Developing nanoscale platforms for localized, sustained magnesium release is promising this approach may improve antimineralization efficacy and reduce systemic exposure.

At the same time, pyrophosphate (PPi) has emerged as a potent physiological inhibitor of calcium phosphate nucleation and crystal growth. Functionalization of chitosan (CS) with pyrophosphate enhances its affinity for calcium and phosphate ions, thereby stabilizing the local microenvironment and preventing hydroxyapatite formation.^10–12^ Chitosan, a cationic polysaccharide derived from chitin, is widely recognized for its chelating capacity, biodegradability, and intrinsic biocompatibility.^10,11^ When combined with PPi, the resulting CS-PPi complex forms nanosized polyelectrolyte structures that prolong PPi activity, which would otherwise be rapidly degraded by alkaline phosphatase.^12^ Such complexes have shown potential for biomineralization control and may serve as building blocks for advanced anticalcification systems.

In parallel, magnesium hydroxide (Mg­(OH)_2_) nanoparticles encapsulated within alginate (Alg) matrices show significant potential for therapeutic applications due to their biocompatibility and controlled ion-release properties.^13,14^ Alginate, a naturally occurring anionic polysaccharide, forms hydrogel-like networks. These networks can encapsulate inorganic particles, improve colloidal stability, and prevent aggregation.^13^ The Mg­(OH)_2_-Alg combination enables sustained Mg^2+^ release. This attenuates pro-calcific signaling, oxidative stress, and inflammatory responses in vascular cells.^14,15^ Furthermore, these systems exhibit low cytotoxicity and high stability, which align well with the requirements for biomedical applications.^5,14^

The dual strategy combining CS-PPi and Mg­(OH)_2_-Alg nanoparticles represents an innovative approach to counteract VC through complementary mechanisms. Specifically, PPi prevents the nucleation and maturation of calcium phosphate crystals,^6^ while Mg^2+^ interferes with crystal growth and osteogenic signaling.^7–9,14^ By integrating these two effects within stable, biocompatible nanostructures, it may be possible to pave the way for localized, injectable antimineralization therapies with minimal adverse effects. Nevertheless, it is important to note that comparative analyses evaluating the physicochemical properties and biocompatibility of both systems under identical conditions remain scarce.

In this work, two nanoparticle systems were designed and compared: chitosan-sodium pyrophosphate (NPs-CS-PPi) and magnesium hydroxide-alginate (NPs-Mg­(OH)_2_-Alg). Both were synthesized using ionic gelation and hydrothermal precipitation, respectively, to achieve stable dispersions with controlled size and surface properties. The nanoparticles were characterized by dynamic light scattering (DLS), Fourier transform infrared spectroscopy (FTIR), thermogravimetric analysis (TGA), X-ray photoelectron spectroscopy (XPS), and electron microscopy (SEM). Their cytocompatibility was assessed using the MTT assay with L929 fibroblasts, in accordance with ISO 10993-5 guidelines. The overall goal was to elucidate structure–property–biocompatibility correlations and to identify the most promising formulation for the future development of injectable, antimineralization therapies targeting vascular calcification.^16,17^

## 2 Experimental Section

### 2.1 Materials

Chitosan (CS, medium molecular weight, 75–85% deacetylation), sodium pyrophosphate (Na_4_P_2_O_7_·10H_2_O), magnesium chloride hexahydrate (MgCl_2_·6H_2_O), sodium hydroxide (NaOH), and sodium alginate (Alg, from brown algae) were obtained from Sigma-Aldrich and used as received. Acetic acid (≥99.7%) and absolute ethanol (≥99.5%) were purchased from Synth (Brazil). Deionized water (resistivity ≥18.2 MΩ·cm) was used in all procedures. The murine fibroblast cell line L929 (ATCC NCTC clone 929 [L cell, CCL-1]) was used for cytotoxicity assays. Dulbecco’s modified Eagle medium (DMEM), fetal bovine serum (FBS), and antibiotic–antimycotic solutions were supplied by Gibco (Thermo Fisher Scientific).

### 2.2 Synthesis of Chitosan-Sodium Pyrophosphate Nanoparticles (NPs-CS-PPi)

NPs-CS-PPi were prepared by a combined ionic gelation and thermal gelation method adapted from Kiilll et al.^12^ Chitosan was dissolved at 10.0 mg mL^–1^ in 0.75% (v/v) acetic acid under ultrasonic treatment for 10 min, followed by magnetic stirring for 24 h at room temperature. Separately, a 10.0 mg mL^–1^ sodium pyrophosphate (PPi) solution was prepared in deionized water.

For nanoparticle formation, 2.0 mL of the CS solution was diluted with 8.0 mL of water and heated to 70–75 °C under continuous stirring to promote partial chain expansion. Simultaneously, 600 μL of the PPi solution was diluted in 5.0 mL of deionized water and added dropwise to the chitosan solution under vigorous stirring at 70–75 °C. The reaction mixture was then stirred continuously for an additional 24 h to allow complete stabilization of the polyelectrolyte complex.

The colloidal suspension was centrifuged at 12,000 rpm for 2 h, and the solid fraction was redispersed in ultrapure water using probe sonication to ensure homogeneity. Both centrifuged and noncentrifuged fractions were freeze-dried for subsequent characterization.

### 2.3 Synthesis of Magnesium Hydroxide-Alginate Nanoparticles (NPs-Mg­(OH)_2_-Alg)

NPs-Mg­(OH)_2_-Alg were synthesized by controlled precipitation followed by surface coating with alginate, as modified from Jiang et al.^13^ and Nakamura et al.^14^

A 1.0 g portion of MgCl_2_·6H_2_O was dissolved in 50 mL of deionized water, and 0.5 g of NaOH was dissolved in 32 mL of water. The MgCl_2_ solution was added dropwise into the NaOH solution under stirring (500 rpm) at ambient temperature, inducing the formation of Mg­(OH)_2_ nuclei. The reaction mixture was heated to 50 °C for 4 h and then subjected to hydrothermal treatment at 150 °C for 2 h in a sealed reactor to promote crystallization.

The resulting suspension was cooled, washed three times with absolute ethanol (centrifugation at 12,000 rpm, 10 min per cycle), and dried under vacuum at 80 °C for 4 h. The dried Mg­(OH)_2_ powder was redispersed in water (0.2 mg mL^–1^) by probe sonication, and a separate alginate solution (0.2 mg mL^–1^) was prepared under slow stirring for 24 h.

The Mg­(OH)_2_ suspension was added dropwise to the alginate solution at a 1:4 (NPs/Alg) mass ratio under constant stirring. The mixture was sonicated again to enhance interfacial interactions and then allowed to equilibrate for 24 h. The coated nanoparticles were recovered by centrifugation, redispersed in ultrapure water, and freeze-dried for characterization.

### 2.4 Characterization

Hydrodynamic diameter, polydispersity index (PDI), and zeta potential (ζ) were measured in triplicate at 25 °C using a Zetasizer Nano ZS instrument (Malvern Instruments, UK) equipped with a 633 nm He–Ne laser and a 173° detection angle.^15^ Samples were dispersed in ultrapure water and filtered through 0.45 μm membranes prior to analysis. The Smoluchowski model was used to calculate zeta potential. Fourier transform infrared (FTIR) spectra were collected on a Cary 630 spectrometer (Agilent) across 4000–500 cm^–1^, with a 4 cm^–1^ resolution and 16 scans per sample. NPs-CS-PPi were analyzed in KBr pellets (1:100 m/m), while NPs-Mg­(OH)_2_-Alg were examined using attenuated total reflectance (ATR). Thermogravimetric analysis (TGA) was used to assess thermal stability and degradation profiles using a NETZSCH STA 449 F3 Jupiter instrument. Approximately 10 mg of lyophilized sample was heated from 30 to 1000 °C at a rate of 10 °C min^–1^ under a synthetic air flow (60 mL min^–1^). Surface chemical composition and bonding states were analyzed by X-ray photoelectron spectroscopy (XPS) (Thermo Fisher Scientific K-Alpha^+^ spectrometer) using Al Kα radiation (1486.6 eV). The analysis chamber pressure was maintained at ∼10^–7^ Pa. Spectra were fitted using CASA XPS software, employing Lorentzian–Gaussian peaks and Shirley background subtraction.^16^ Morphology and particle size were examined using a Mira 3 Tescan field-emission scanning electron microscope (FEG-SEM) operated in transmission SEM (TSEM) mode at an accelerating voltage of 30 kV. Energy-dispersive X-ray spectroscopy (EDS, Bruker) was used for elemental analysis. In vitro cytotoxicity assay and cytocompatibility were evaluated using the MTT assay in accordance with ISO 10993-5:2009. L929 cells were cultured in DMEM supplemented with 10% FBS and 1% antibiotic–antimycotic solution at 37 °C under a 5% CO_2_ atmosphere. Cells (1 × 10^4^ cells per well) were seeded into 96-well plates and incubated for 12 h for attachment. Nanoparticle suspensions prepared in phenol-red-free DMEM were added at concentrations of 1%, 2.5%, 5%, 7.5%, and 10% (v/v) and incubated for 24 h. Untreated cells and cells treated with 10% DMSO served as negative and positive controls, respectively. After exposure, the medium was replaced with 1 mg mL^–1^ MTT solution and incubated for 3 h. The resulting formazan crystals were dissolved in 100 μL DMSO, and absorbance was measured at 570 nm using a microplate reader (BioTek). Cell viability was expressed as a percentage relative to control.^17^ Data were reported as mean ± standard deviation (SD) of seven technical replicates. Statistical analyses were performed using the Shapiro–Wilk test for normality, followed by one-way ANOVA and Dunnett’s post hoc test (*p* < 0.05).

## 3 Results and Discussion

Figure 1 schematically illustrates the synthesis pathways and proposed antimineralization mechanisms of the two nanoparticle systems. Chitosan–sodium pyrophosphate nanoparticles (NPs-CS-PPi) were prepared via ionic and thermal gelation, in which protonated amino groups of chitosan (–NH_3_^+^) interact electrostatically with pyrophosphate anions (P_2_O_7_^4–^), leading to the formation of stable, positively charged nanostructures. In contrast, magnesium hydroxide-alginate nanoparticles (NPs-Mg­(OH)_2_-Alg) were obtained by controlled precipitation followed by alginate coating, yielding negatively charged particles with enhanced colloidal stability. The combination of biopolymers such as chitosan and alginate has been extensively explored in the design of hybrid nanoparticles due to their ability to form stable colloidal systems with tunable physicochemical properties for biomedical applications.^18^ The schematic also summarizes dual complementary antimineralization mechanisms: (i) pyrophosphate inhibits calcium phosphate nucleation by chelating Ca^2+^ ions and preventing hydroxyapatite seed formation, and (ii) Mg^2+^ ions released from Mg­(OH)_2_ interfere with crystal growth and promote the formation of less stable amorphous phases. By acting at distinct stages of hydroxyapatite formation, these mechanisms may collectively contribute to the prevention of vascular mineralization; however, further studies are required to experimentally validate these proposed mechanisms.^16^

**Figure 1:**
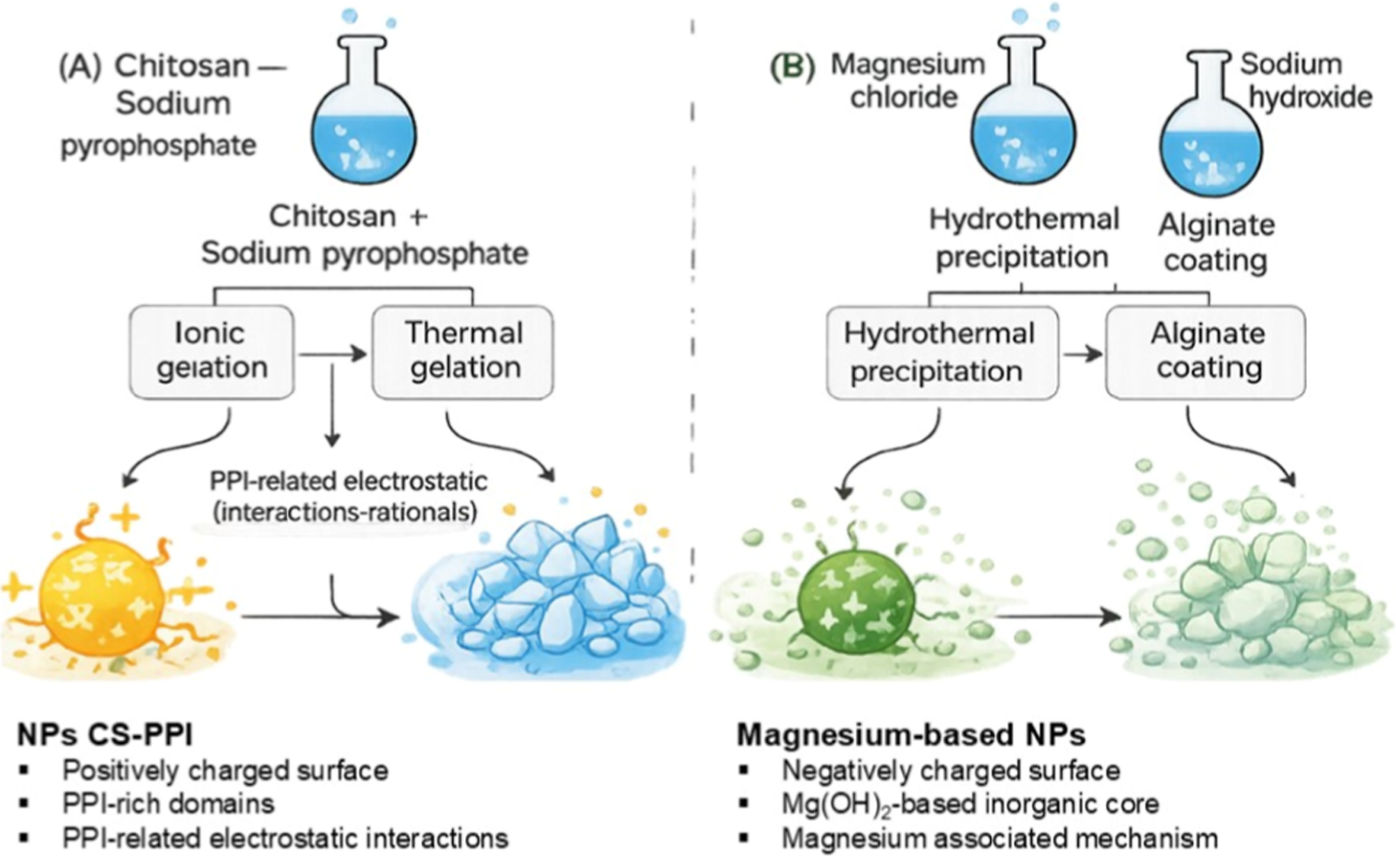
Schematic illustration of the synthesis routes and key physicochemical features of (A) chitosan-sodium pyrophosphate (NPs-CS-PPi) and (B) magnesium hydroxide-alginate (NPs-Mg­(OH)_2_-Alg) nanoparticles. The schemes depict the respective preparation pathways, including ionic and thermal gelation for NPs-CS-PPi and controlled precipitation followed by alginate coating for NPs-Mg­(OH)_2_-Alg, along with determined surface properties and literature-based mechanistic rationales.

### 3.1 Morphology, Particle Size Distribution, and Surface Charge

Dynamic light scattering (DLS) and zeta potential (ζ) analyses confirmed the formation of stable nanoscale dispersions for both formulations. The particle size distributions from DLS are in Figure S1 (Supporting Information). NPs-CS-PPi showed an average hydrodynamic diameter of 185 ± 12 nm with a polydispersity index (PDI) of 0.28 ± 0.03. NPs-Mg­(OH)_2_-Alg had a slightly larger mean size of 215 ± 18 nm and a PDI of 0.31 ± 0.04. These results indicate monomodal size distributions and moderate polydispersity suitable for biomedical applications.^15^

The ζ potential values revealed distinct interfacial behaviors: NPs-CS-PPi presented a positive charge (+32.6 ± 1.8 mV) due to the protonated amino groups of chitosan, whereas NPs-Mg­(OH)_2_-Alg showed a negative potential (−35.4 ± 2.1 mV), consistent with alginate’s carboxylate functionality. The opposite surface charges suggest different cell-interaction mechanisms and colloidal stabilization pathways, electrostatic repulsion for Alg-coated particles and charge shielding for CS-based ones.

Scanning electron microscopy images obtained in transmission mode (TSEM) revealed predominantly spherical to near-spherical morphologies with smooth surfaces for both nanoparticle systems, indicating homogeneous particle formation. Additional high-magnification TSEM micrographs are provided in Figure S2 (Supporting Information). NPs-CS-PPi displayed a homogeneous distribution of smaller particles forming soft agglomerates, while NPs-Mg­(OH)_2_-Alg exhibited more defined crystalline edges, reflecting the partial retention of the Mg­(OH)_2_ lattice within the polymeric matrix. These morphological differences are consistent with the ionic cross-linking density and hydrothermal crystallization steps.^13,14^

### 3.2 Structural, Chemical, and Thermal Characterization

Fourier-transform infrared spectroscopy (FTIR) confirmed successful formation of both nanocomposites (Figure 2a,b). In NPs-CS-PPi, characteristic CS bands were observed at 3445 cm^–1^ (–OH and –NH_2_ stretching), 1642 cm^–1^ (amide I), and 1556 cm^–1^ (amide II), while the new absorption at 1190 and 1060 cm^–1^ corresponds to the asymmetric and symmetric stretching of P–O–P bonds, confirming the electrostatic complexation between chitosan and pyrophosphate.^12^

**Figure 2:**
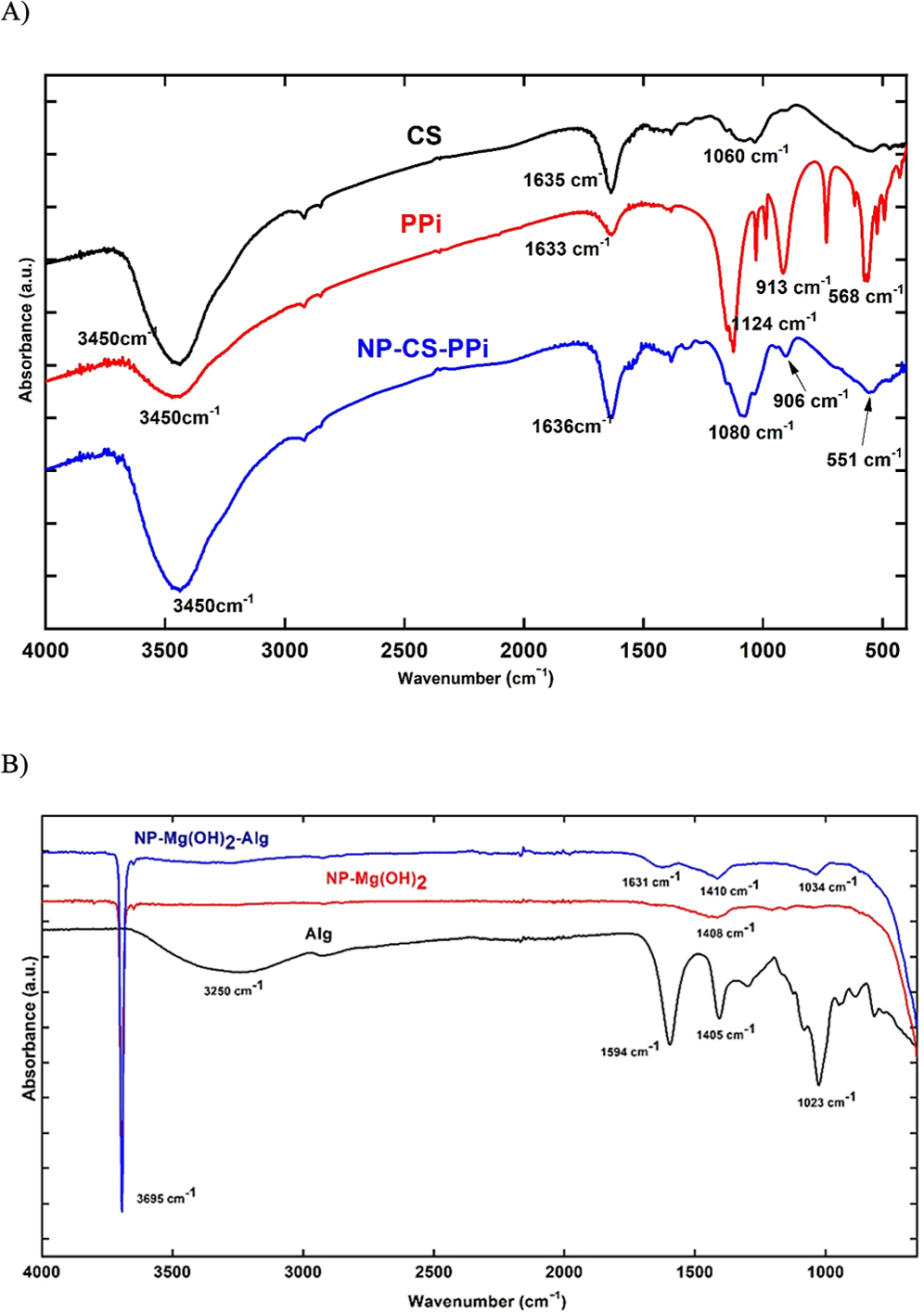
FTIR spectra of (a) CS, PPi, and NPs-CS-PPi; and (b) Alg, Mg­(OH)_2_, and NPs-Mg­(OH)_2_-Alg. Characteristic absorption bands confirm P–O–P and COO^–^–Mg^2+^ interactions, evidencing polymer–inorganic hybrid formation.

In the case of NPs-Mg­(OH)_2_-Alg, strong bands at 3440 cm^–1^ (O–H stretching), 1635 cm^–1^ (COO^–^ asymmetric stretching), and 1415 cm^–1^ (COO^–^ symmetric stretching) indicate alginate coordination with magnesium ions. A weak band near 3690 cm^–1^, attributed to Mg–OH lattice vibration, confirmed the presence of crystalline Mg­(OH)_2_ within the alginate matrix.^13^ These spectral features indicate that ionic interactions between the organic and inorganic phases play a key role in stabilizing both hybrid systems (Figure 2).

X-ray photoelectron spectroscopy (XPS) was used to clarify the chemical states of elements in both nanocomposite systems (Figures 3 and 4). For NPs-CS-PPi (Figure 3), the C 1s spectrum showed three components at 284.8 eV (C–C/C–H), 286.3 eV (C–O/C–N), and 287.9 eV (C=O), all characteristic of the chitosan backbone. Peaks in the O 1s region at 531.4 eV (P–O) and 532.8 eV (C–O–C) confirmed the incorporation of a pyrophosphate group. The N 1s signal at 399.5 eV was attributed to protonated amino species (–NH_3_^+^), indicating electrostatic interactions with phosphate moieties. The P 2p doublet at 133.6 eV indicated P=O and P–O–P bonds from pyrophosphate anions. This shows a stable ionic complex formed between the cationic chitosan matrix and the anionic phosphate species. These results confirm that CS-PPi nanoparticles are chemically cross-linked hybrids, stabilized by amine-phosphate electrostatic complexation.

**Figure 3:**
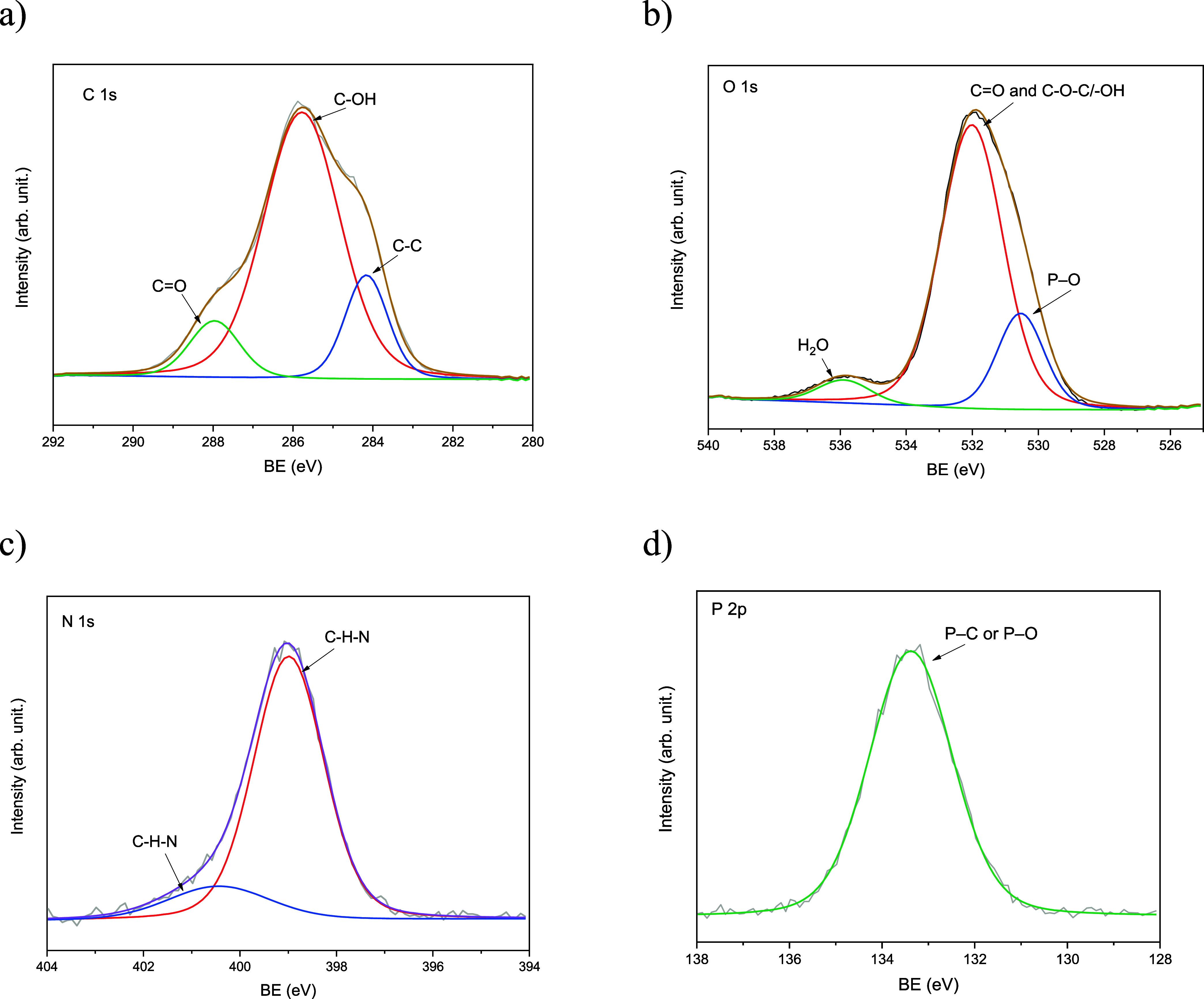
XPS spectra of NPs-CS-PPi showing (a) C 1s, (b) O 1s, (c) N 1s, and (d) P 2p regions. Deconvoluted peaks correspond to C–O, C–N, and P–O bonds, confirming electrostatic complexation between protonated amine groups of chitosan and pyrophosphate anions.

**Figure 4:**
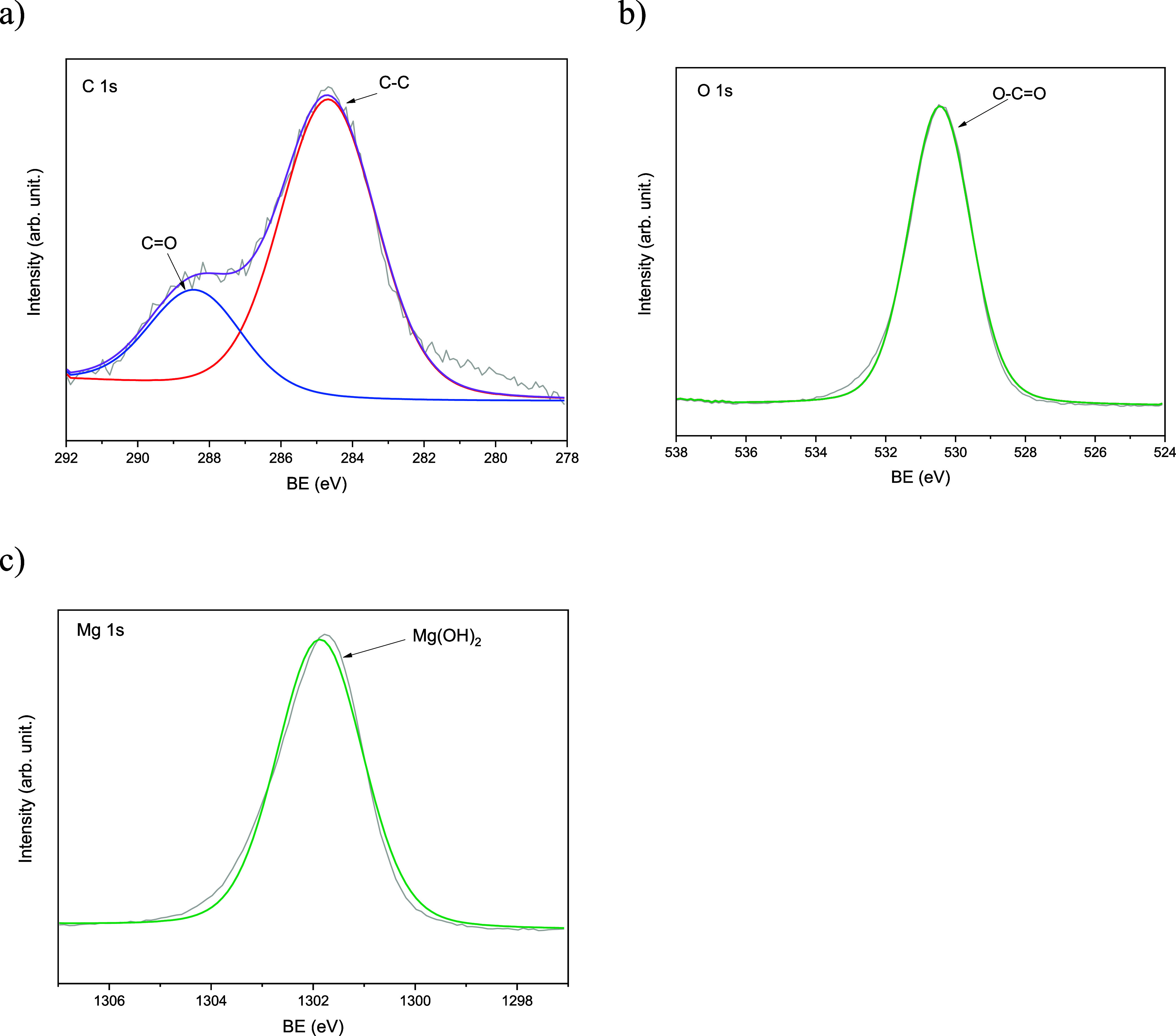
XPS spectra of NPs-Mg­(OH)_2_-Alg showing (a) C 1s, (b) O 1s, and (c) Mg 2p regions.

In the NPs-Mg­(OH)_2_-Alg (Figure 4), the C 1s spectrum contained peaks at 284.8 eV (C–C/C–H), 286.3 eV (C–O), and 288.4 eV (O–C=O), consistent with the presence of alginate carboxylate groups. The O 1s region displayed a major component at 530.8 eV, associated with Mg–O bonding, and a secondary contribution at 532.3 eV, attributed to C–O groups. The Mg 2p feature at 50.4 eV corresponds to Mg­(OH)_2_, confirming that magnesium remains predominantly in the hydroxide form after alginate coating. These spectral assignments demonstrate strong ionic coordination between the carboxylate (–COO^–^) groups of alginate and Mg^2+^ centers, validating the formation of a uniform Mg­(OH)_2_-Alg hybrid interface.

Overall, the XPS results complement the FTIR data by confirming the coexistence of polymeric (C–O, C–N, C=O) and inorganic (P–O, Mg–O) chemical environments and supporting effective surface functionalization, which is associated with improved aqueous stability of the nanoparticles. The presence of O–C=O and Mg–O groups confirms coordination between alginate carboxylate groups and Mg­(OH)_2_, indicating the formation of an effective polymer–inorganic hybrid.

Thermogravimetric analysis (TGA) revealed distinct thermal degradation profiles for the two systems (Figure 5a,b). NPs-CS-PPi exhibited a two-step weight loss, with an initial loss of approximately (∼10%) below 120 °C attributed to moisture removal, followed by a major loss (∼60%) between 250 and 380 °C associated with CS backbone degradation and phosphate condensation.^12^ Conversely, NPs-Mg­(OH)_2_-Alg displayed three degradation regions: (i) water evaporation at 120 °C, (ii) decomposition of the organic alginate component between 220 and 350 °C, and (iii) dehydroxylation of Mg­(OH)_2_ between 380 and 450 °C. The higher residual mass (∼35%), corresponding to thermally stable MgO, indicates greater thermal stability compared to NPs-CS-PPi.

**Figure 5:**
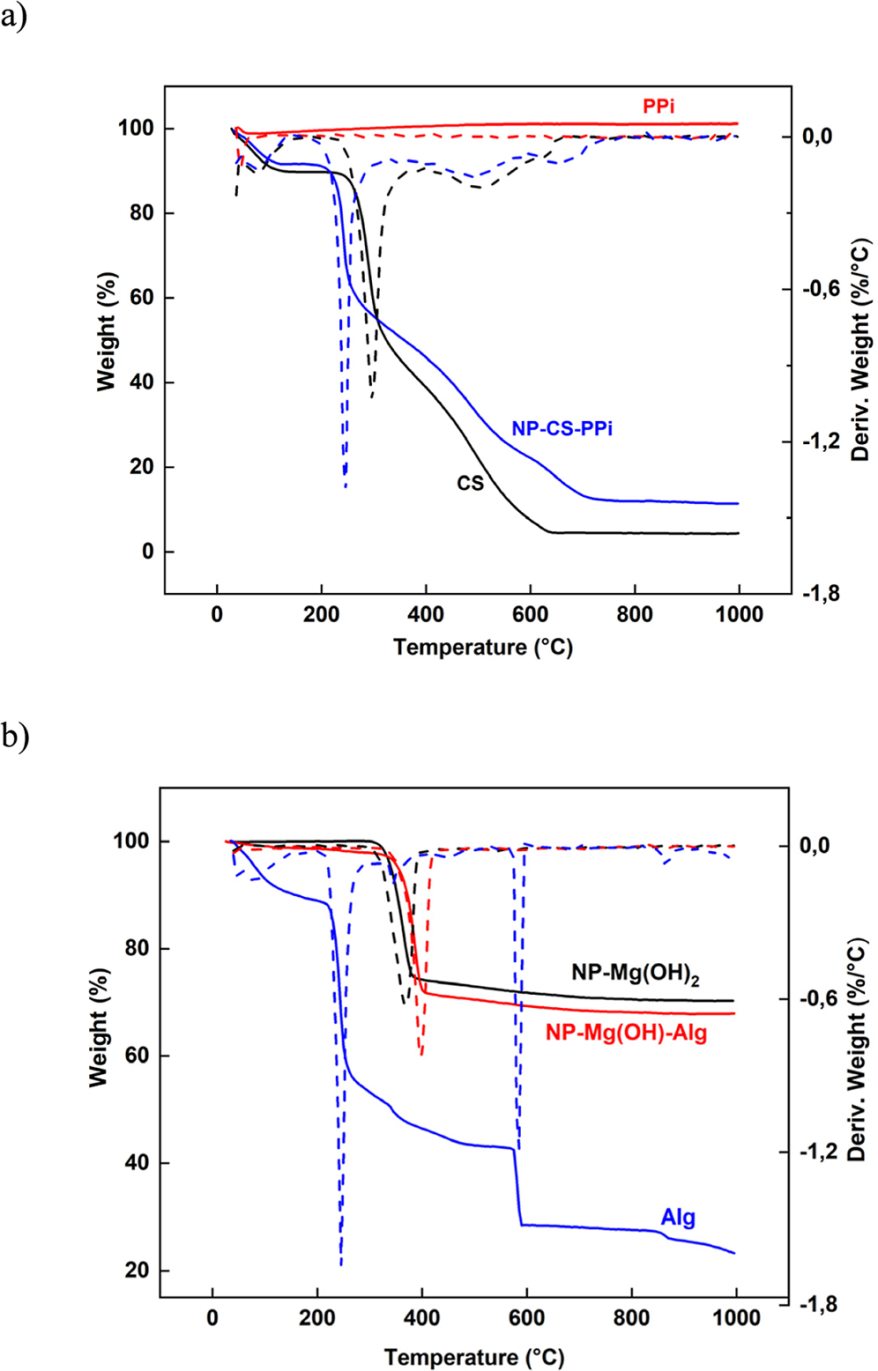
Thermogravimetric analysis (TGA) and derivative thermogravimetric (DTG) curves of (a) chitosan (CS), sodium pyrophosphate (PPi), and NPs-CS-PPi, and (b) alginate (Alg), magnesium hydroxide (Mg­(OH)_2_), and NPs-Mg­(OH)_2_-Alg. The DTG curves highlight the main thermal degradation events of the polymeric components and nanoparticle formulations.

### 3.3 In Vitro Cytocompatibility

The cytotoxicity of both systems was assessed using a MTT assay with L929 fibroblasts (Figure 6). After 24 h of exposure, cell viability remained above 85% for NPs-CS-PPi and 92% for NPs-Mg­(OH)_2_-Alg at concentrations up to 10% v/v, indicating noncytotoxic profiles according to ISO 10993-5.^19^ The slightly higher viability with Mg-based nanoparticles suggests superior biocompatibility, likely due to alginate’s mild surface chemistry and controlled Mg^2+^ release, which mitigate local pH shifts. As shown in Figure 6, both formulations-maintained viability above 85% across all tested concentrations, confirming their cytocompatibility.

**Figure 6:**
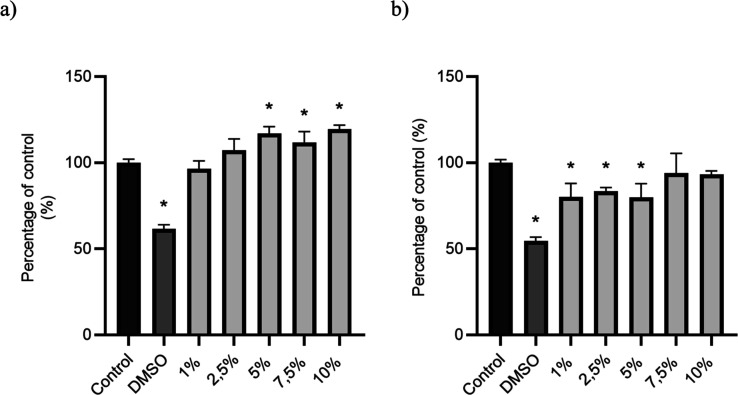
(a) Cytotoxicity analysis of L929 cells after 24 h of exposure to NPs-Mg­(OH)_2_-Alg at different concentrations. *p < 0.05 vs control; (b) cytotoxicity analysis of L929 cells after 24 h of exposure to NPs-CS-PPi at different concentrations. *p < 0.05 vs control.

These results confirm that both formulations are suitable for biomedical use, with NPs-Mg­(OH)_2_-Alg demonstrating enhanced cellular tolerance. This is consistent with the literature, which shows that magnesium-containing biomaterials promote cellular adhesion and metabolic activity.^7,8^ Similar effects have been reported for magnesium-based systems incorporated into bioresorbable vascular scaffolds, which enhance endothelial viability and reduce inflammatory responses both in vitro and in vivo.^20^

### 3.4 Comparative Evaluation and Biomedical Relevance

To highlight physicochemical and biologiacal differences between the two nanoparticle systems, Table 1 summarizes their key experimentally determined properties. NPs-CS-PPi exhibited a smaller average particle size and a higher positive surface charge, characteristics that favor electrostatic interactions with negatively charged biological components and phosphate species. In contrast, NPs-Mg­(OH)_2_-Alg, showed enhanced colloidal and thermal stability, along with higher cell viability, indicating a potentially safer profile for further biomedical evaluation.

**Table 1:** Comparative Physicochemical and Biological Characteristics of the NPs-CS-PPi and NPs-Mg­(OH)_2_-Alg

property	NPs-CS-PPi	NPs-Mg(OH)_2_-Alg
hydrodynamic diameter (nm)	185 ± 12	215 ± 18
PDI	0.28 ± 0.03	0.31 ± 0.04
zeta potential (mV)	+32.6 ± 1.8	–35.4 ± 2.1
thermal degradation onset (°C)	250	380
cell viability (% at 10% v/v)	85 ± 3	92 ± 2
residual mass (%)	22	35
stability in aqueous media	moderate	high

Although no direct mineralization inhibition assays were performed in this study, the distinct compositions of the nanoparticle systems support different proposed antimineralization mechanisms based on established literature. Pyrophosphate is known to act as a nucleation inhibitor of calcium phosphate phases, while magnesium ions interfere with crystal growth and stabilize amorphous phases.^7,9^ These mechanistic considerations provide a rationale for future studies employing dedicated vascular calcification models.

Biomimetic polyphosphate structures have been shown to inhibit multiple stages of pathological crystallization, supporting the effectiveness of phosphate-rich systems such as pyrophosphate in blocking mineral nucleation.^21^ Future work may explore the encapsulation of anti-inflammatory or osteogenic-modulating agents (e.g.,fetuin-A mimetics or bisphosphonates) within these nanoparticles to enhance therapeutic outcomes.

### 3.5 Discussion and Application Perspective

The physicochemical characterization and short-term cytocompatibility results presented herein establish a comparative hybrid nanoparticle platform composed of chitosan-pyrophosphate (NPs-CS-PPi) and magnesium hydroxide-alginate (NPs-Mg­(OH)_2_-Alg) systems. Given the absence of previous reports describing the synthesis and application of these specific hybrid formulations in the context of vascular calcification, this work primarily provides a foundational material-science framework for subsequent biological and translational investigation.

Chitosan has emerged as a versatile biopolymer for drug delivery applications due to its intrinsic biocompatibility, biodegradability, cationic character, and chemical tunability.^22^ Advances in multidimensional and 4D printing technologies have further demonstrated the potential of chitosan-based systems for spatially controlled and stimuli-responsive delivery platforms. These developments underscore the adaptability of chitosan matrices in modulating mechanical stability, ion-binding capacity, and release kinetics. The present nanosystems extend this material versatility toward mineralization-modulating applications, although their performance in vascular calcification remains to be functionally validated.

Magnesium and pyrophosphate were incorporated based on established evidence describing their roles in calcium–phosphate homeostasis.^16,23^ PPi is a potent endogenous inhibitor of hydroxyapatite nucleation and crystal propagation, acting through high-affinity binding to nascent crystal surfaces.^24^ Complete inhibition of hydroxyapatite formation has been reported at micromolar PPi concentrations, levels substantially lower than physiological calcium or phosphate concentrations. Under hyperphosphatemic conditions, such as those observed in chronic kidney disease, physiological PPi levels may become insufficient to prevent pathological mineral deposition, supporting the concept of controlled PPi supplementation.

Magnesium exerts complementary effects by interfering with crystal lattice organization and modulating osteogenic signaling pathways. Notably, Mg^2+^ exhibits concentration- and stage-dependent biological behavior. In vitro studies suggest that concentrations in the range of 2–10 mM may be biologically favorable, whereas excessive or prolonged exposure can impair later-stage mineral maturation.^25^ This concentration sensitivity highlights the importance of precisely controlled Mg^2+^ release kinetics. Emerging concepts such as hierarchical therapeutic ion release systems, developed in regenerative medicine, demonstrate that temporally regulated ion delivery can enhance biological outcomes compared to uniform sustained release. Although vascular calcification differs from bone regeneration, the principle of stage-adapted ion modulation may be relevant for controlling vascular smooth muscle cell phenotype and mineral nucleation dynamics.

Route of administration represents a critical determinant in nanoparticle design and future development. Intravenous application, which aligns with the systemic nature of vascular calcification, would require rigorous evaluation of colloidal stability in physiological media, protein adsorption behavior, hemocompatibility, biodistribution, and clearance kinetics. Surface charge of +32.6 mV for NPs-CS-PPi and −35.4 mV for NPs-Mg­(OH)_2_-Alg may significantly influence plasma interactions and mononuclear phagocyte system uptake. In addition, systemic magnesium regulation must be carefully considered, particularly in patients with impaired renal function.^7^

Localized administration strategies could reduce systemic exposure but would necessitate optimization of nanoparticle retention, degradation behavior, and localized ion release profiles. The nanoscale size distribution and defined zeta potential values observed for both formulations are compatible with route-specific optimization. Nevertheless, release kinetics under physiologically relevant conditions and long-term biological response require dedicated evaluation.

Cytocompatibility was assessed using an MTT assay with L929 fibroblasts, with nanoparticle suspensions prepared in phenol-red-free DMEM and tested at concentrations of 1–10% (v/v). Cell viability remained above 85% for NPs-CS-PPi and 92% for NPs-Mg­(OH)_2_-Alg, classifying both systems as noncytotoxic. Although cytocompatibility was evaluated using nanoparticle suspensions at 1–10% (v/v), the effective concentration of released PPi is expected to be substantially lower than the total nanoparticle volumetric fraction. Given that hydroxyapatite inhibition has been reported at micromolar PPi concentrations, more than 1000-fold lower than physiological calcium or phosphate levels, even partial ion release may reach biologically relevant thresholds, depending on encapsulation efficiency and release kinetics.

It should be emphasized, however, that short-term in vitro cytocompatibility does not allow determination of maximum tolerated dose (MTD). MTD assessment requires structured in vivo dose-escalation studies evaluating systemic toxicity, electrolyte balance, renal and hepatic function, and histopathology. Furthermore, standardized protocols for ion release testing, considering sample-to-medium ratio, medium composition, and dynamic conditions, are essential to correlate in vitro concentrations with in vivo exposure.

Similarly, although the mechanistic basis for PPi- and Mg^2+^-mediated inhibition of pathological mineralization is well established in the literature, functional mineralization assays have not yet been performed for these specific hybrid nanosystems. Given the novelty of the formulations reported here, the primary objective was to establish reproducible synthesis, structural stability, and preliminary cytocompatibility. Future studies will need to include quantitative mineralization assays, such as calcium–phosphate precipitation models or vascular smooth muscle cell calcification systems, to validate antimineralization efficacy under hyperphosphatemic conditions.

## 4 Conclusions

This work synthesized, characterized, and comparatively evaluated two hybrid nanoparticle systems: chitosan-sodium pyrophosphate (NPs-CS-PPi) and magnesium hydroxide-alginate (NPs-Mg­(OH)_2_-Alg). The applied synthesis strategies ionic gelation, controlled precipitation, and polymer coating yielded stable colloidal dispersions with distinct surface chemistry and physicochemical profiles.

Spectroscopic and thermal analyses confirmed the formation of integrated polymer–inorganic hybrid architectures. NPs-Mg­(OH)_2_-Alg exhibited enhanced thermal stability associated with the inorganic phase, whereas NPs-CS-PPi displayed characteristics consistent with phosphate-enriched domains. Surface charge measurements reflected the distinct polymer matrices, with positively charged CS-based nanoparticles and negatively charged alginate-coated systems.

Short-term cytocompatibility assays using L929 fibroblasts demonstrated noncytotoxic behavior for both formulations, maintaining cell viability above 85% after 24 h of exposure. Slightly higher viability observed for NPs-Mg­(OH)_2_-Alg is consistent with the interfacial characteristics of alginate-based materials.

Overall, this study establishes a comparative structural, interfacial, and cytocompatibility framework for two polymer–inorganic nanosystems prepared under standardized conditions, providing a reference basis for subsequent biological investigation.

## Supplementary Material



## Notes

It includes detailed DLS size distributions (Figure S1), additional SEM images (Figure S2) (PDF)
