# Supplementary material for: Comparative Study of Chitosan-Pyrophosphate and Magnesium Hydroxide-Alginate Hybrid Nanoparticles: Physicochemical Properties and Cytocompatibility toward Vascular Calcification Applications
Source: ACS Omega. 2026 May 1;11(18):26544–52. doi: 10.1021/acsomega.5c12883 (PMC13177163; doi:10.1021/acsomega.5c12883)
Supplement: Supplementary file 1 [file ao5c12883_si_001.pdf]

Supporting Information for:

Comparative Study of Chitosan–Pyrophosphate and Magnesium Hydroxide–Alginate Hybrid Nanoparticles: Physicochemical Properties and Cytocompatibility toward Vascular Calcification Applications

Jorge Costa Silva Filho[1]*, Maíra Maftoum Costa[1], Marcelo de Sousa[2], Laryssa Cristine Ribeiro dos Santos[1], Talita Mazon[1]*

[1]Centro de Tecnologia da Informação Renato Archer, Rodovia D. Pedro I (SP-65), Km 143,6 - Bairro Amarais, Campinas – São Paulo, zip code 13069-901, Brazil. -

[2]Department of Chemistry, University Federal of Amazonas (UFAM). Av. Rodrigo Otávio, 6.200, zip code 69077-000, Manaus, Amazonas, Brazil.

* Corresponding authors: jorge-costa.silva@cti.gov.br and talita.mazon@cti.gov.br

a)

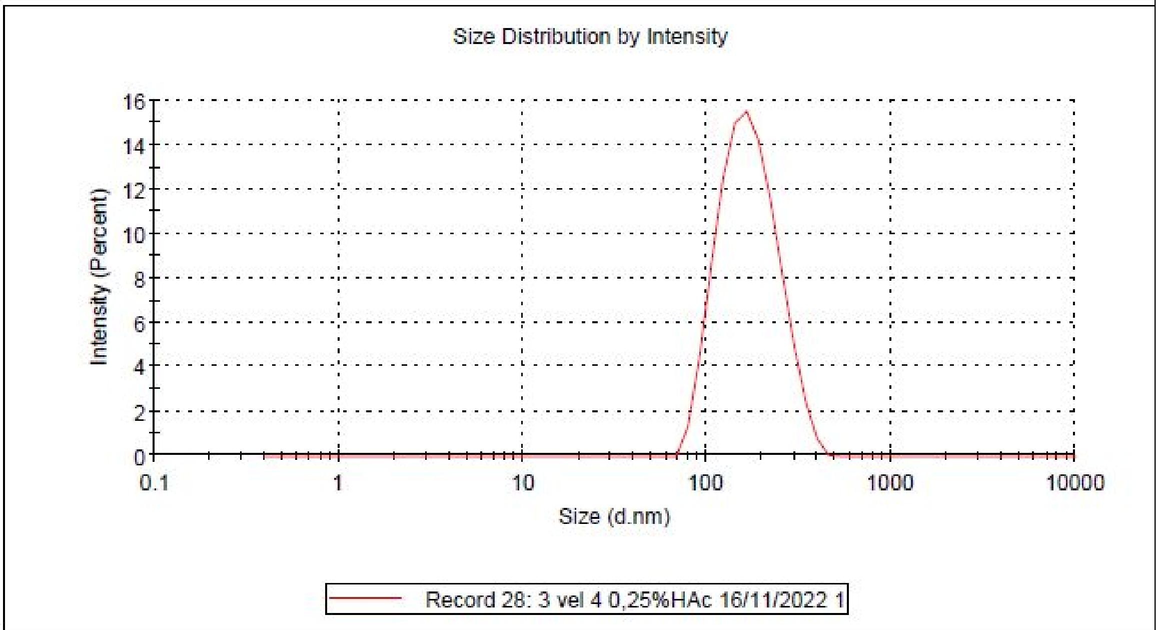

b)

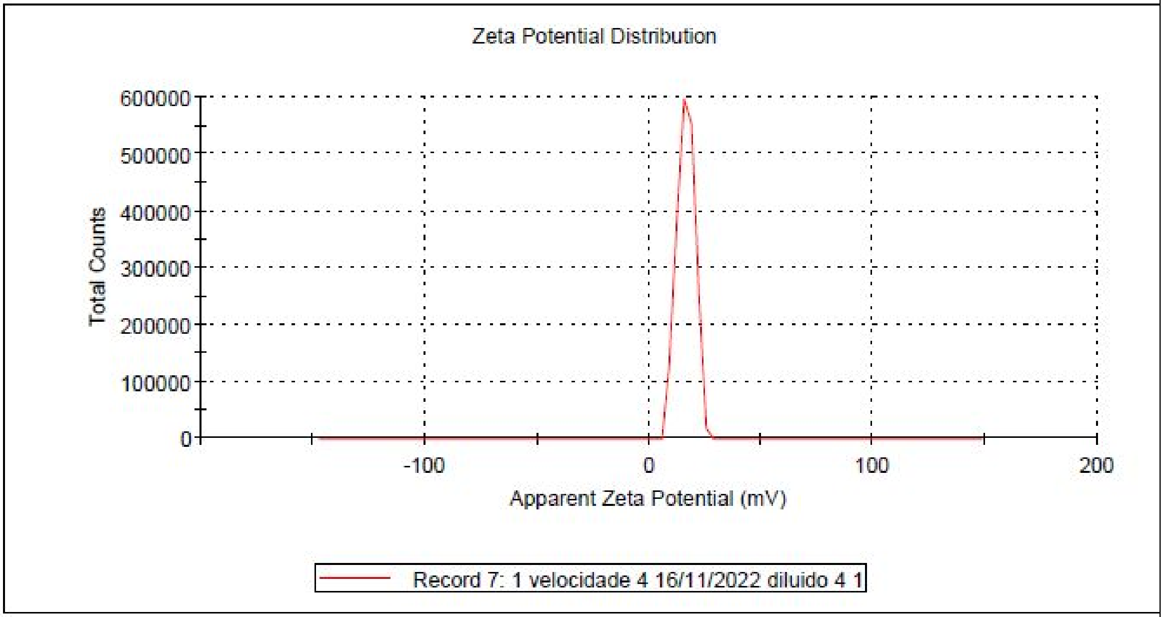
Zeta Potential Distribution
600000
500000
400000
300000
200000
100000
0
Total Counts
-100
0
100
200
Apparent Zeta Potential (mV)
Record 7: 1 velocidade 4 16/11/2022 diluido 4 1

c)

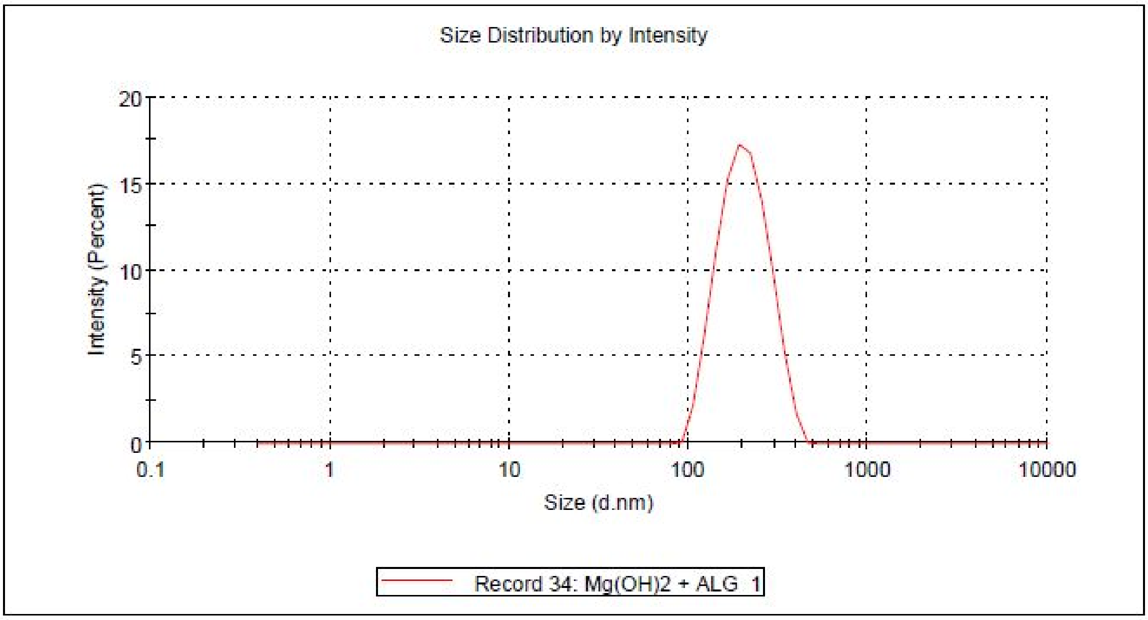
Size Distribution by Intensity
20
15
10
5
0
Intensity (Percent)
0.1
1
10
100
1000
10000
Size (d.nm)
Record 34: Mg(OH)2 + ALG 1

d)

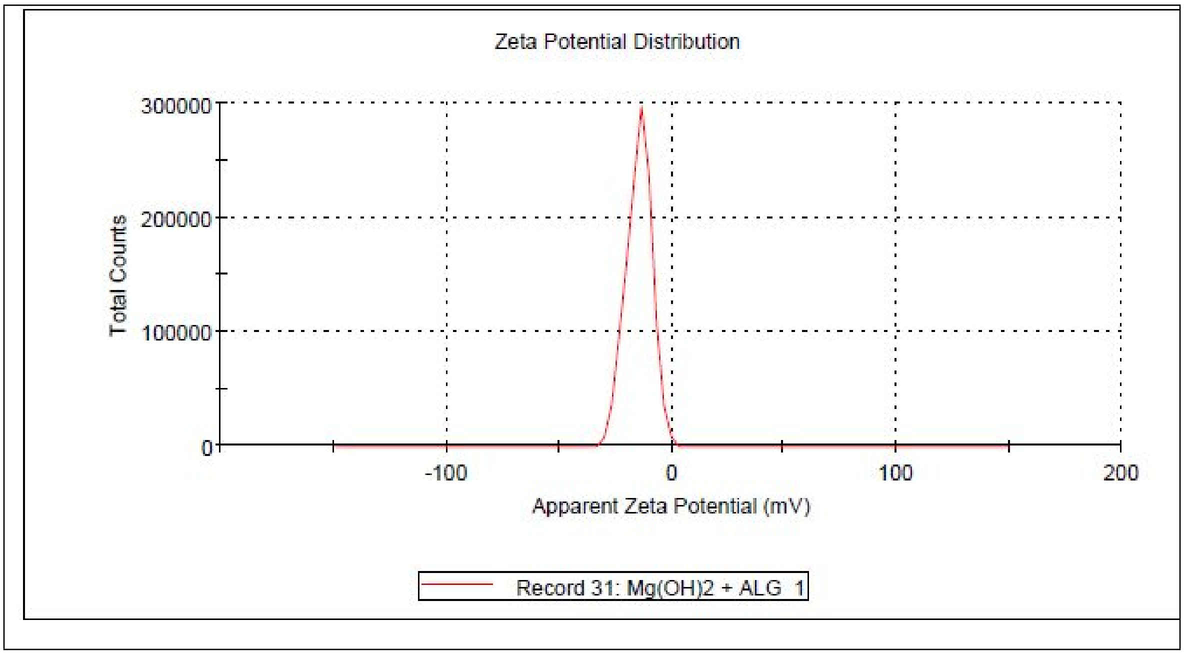

Figure S1. Particle size distribution histograms of (a) NPs-CS-PPi diameter, (b) Zeta potential NPs-CS-PPi, (c) diameter NPs-$Mg(OH)_2$-Alg and (d) NPs-$Mg(OH)_2$-Alg Zeta potential obtained by dynamic light scattering (DLS). Both systems exhibited monomodal size distributions consistent with hydrodynamic diameters reported in the main text.

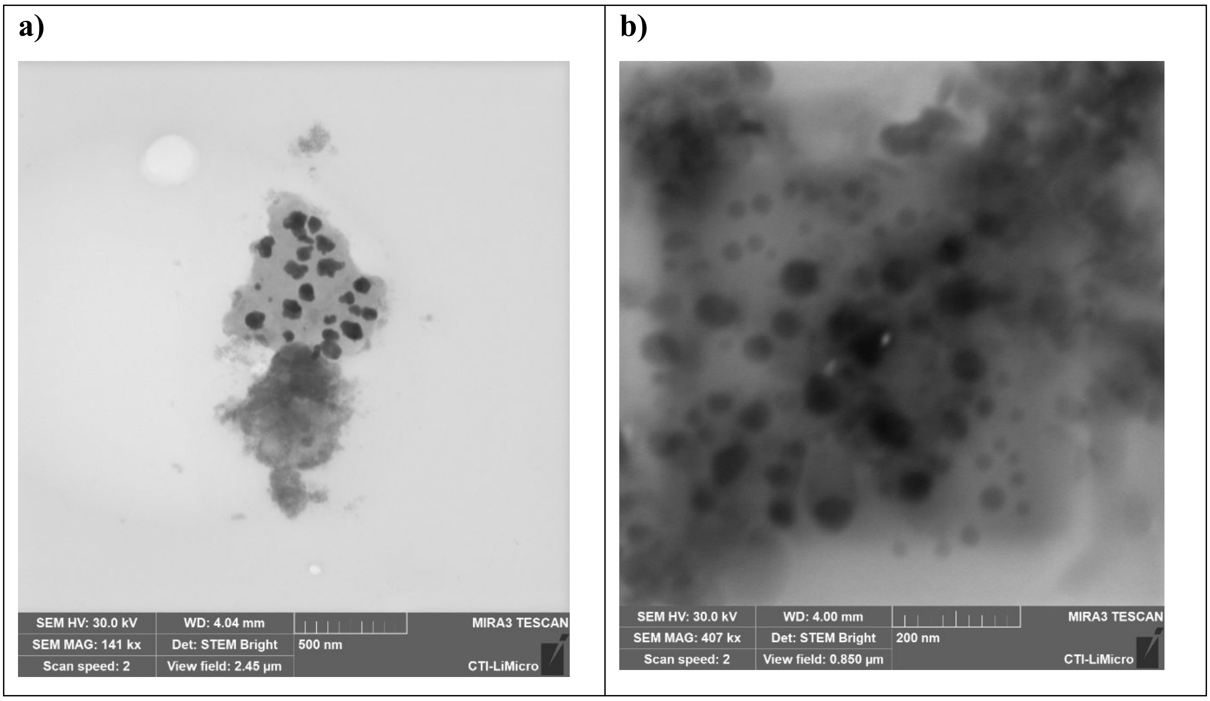

Figure S2. Scanning electron microscopy images obtained in transmission mode (TSEM) of (a) NPs-CS-PPi and (b) NPs-$Mg(OH)_2$-Alg at higher magnification. The particles exhibit near-

spherical morphology and smooth surface texture, consistent with uniform dispersion and hybrid polymer–inorganic structure.
